# Prediction of Biaxial Properties of Elastomers and Appropriate Data Processing

**DOI:** 10.3390/polym16152190

**Published:** 2024-08-01

**Authors:** Jakub Javořík, Rohitha Keerthiwansa, Vladimír Pata, Soňa Rusnáková, Barbora Kotlánová, Michal Grunt, Michal Sedlačík

**Affiliations:** 1 Faculty of Technology, Tomas Bata University in Zlín, 760 01 Zlin, Czech Republic; pata@utb.cz (V.P.); rusnakova@utb.cz (S.R.); b_kotlanova@utb.cz (B.K.); grunt@utb.cz (M.G.); msedlacik@utb.cz (M.S.); 2 Faculty of Technological Studies, Uva Wellassa University, Kelaniya 11600, Sri Lanka; rohitha@uwu.ac.lk

**Keywords:** elastomer, hyperelasticity, uniaxial tension, equibiaxial tension, experimental data, curve processing

## Abstract

An equibiaxial tension test could be necessary to set up hyperelastic material constants for elastomers exactly. Unfortunately, very often, only uniaxial tension experimental data are available. It is possible to use only uniaxial data to compute hyperelastic constants for a hyperelastic model, but the prediction of behavior in different deformation modes (as is equibiaxial or pure shear) will not work correctly with this model. It is quite obvious that there is some relation between uniaxial and equibiaxial behavior for the elastomers. Thus, we could use uniaxial data to predict equibiaxial behavior. If we were able to predict (at least approximately) equibiaxial data, then we could create a hyperelastic model usable for the general prediction of any deformation mode of elastomer. The method of the appropriate processing of experimental data for such prediction is described in the article and is verified by the comparison with the experiment. The presented results include uniaxial and equibiaxial experimental data, the created average curve of both the deformation modes, and the predicted equibiaxial data. Using Student’s *t*-test, a close coincidence of the real and predicted equibiaxial data was confirmed.

## 1. Introduction

The process by which a stress/strain curve of an elastomer is obtained from a set of tested specimens is not usually described in detail in the current scientific works. One of the main motivations of the article is the formulation of a method for the processing of this type of data (curves or chains of values). Such data processing is not only important for obtaining the resulting average curves, but it is equally important if we need to compare the results of several different experiments, especially if we need to use standard statistical tools for testing the significance of the difference in various results. Without this procedure, the results are evaluated and compared only subjectively without any objective criteria and metrics.

The uniaxial tension test only is not sufficient to describe the hyperelastic properties of elastomers appropriately [1,2,3]. Using only uniaxial tension input data, the prediction of the behavior of elastomer in any other deformation modes is uncertain, inexact, and very often completely unrealistic (for example, a prediction of 100 times higher values than the reality or even the prediction of the negative values of stress for the positive values of strain), as published previously in [4,5]. Data from the next two deformation modes (i.e., equibiaxial tension and pure shear [6,7]) are very important to set hyperelastic material constants correctly. The importance of these tests of elastomers was published previously [8,9] and possible misleading errors due to the omission of some deformation modes experiments are described in [4,10]. Nevertheless, in some situations, we have only uniaxial data, and we are not able to obtain the experimental results of the next deformation modes. Even in such cases, it is always better to use this set of accurate uniaxial data together with some other set of non-experimental approximate equibiaxial data than to use the same set of uniaxial data alone (as is demonstrated in [5]). In other words, if only uniaxial data are used, we obtain a hyperelastic material model that describes very accurately behavior in uniaxial tension but is not able to correctly predict any other type of loading. The goal of the research and method described in the article is to be able (from uniaxial data only, again) to obtain a hyperelastic material model that will describe accurately enough and realistically the behavior in uniaxial tension but also in any other type of loading.

If only uniaxial data are available for the hyperelastic model definition, we have to ensure that the prediction of the behavior of the other deformation modes will not be unreal. Such a prediction (i.e., for different deformation modes) will certainly not be absolutely correct but must be within the presumed approximate limits. A method for setting up these limits is described in this article. No such method is defined in the field of the mechanics of elastomers yet, which means that researchers who used only uniaxial experimental data could simulate only uniaxial tension or (for any other type of loading) they had to perform experiments of different deformation modes.

The goal is to find a method to determine relevant hyperelastic parameters that can be used to predict any deformation modes when only uniaxial experimental data are accessible. To achieve this goal, we have to be able not only to test the material correctly but also to process the measured data in an applicable form which is the next important object and benefit of the article.

The basic methods for the testing of elastomers are known and sufficiently described [11,12,13,14,15,16,17]. The outputs of the tests are not discrete values but the whole curves of the stress/strain relation (or rather chains of discrete points). There is no problem in using such data to evaluate hyperelastic model parameters [18,19,20,21,22,23]. But it is quite complicated to compare different data of this kind (for example to evaluate the model suitability) using objective statistical tools to quantify some values describing the size of the difference, the statistical signification of the difference, and so on.

## 2. Materials and Methods

The first important thing to mention is that we do not want to use any form of regression to process the data curves. The reason for this is that these data are measured as inputs for hyperelastic model parameters determination. Therefore, the final result, i.e., the hyperelastic model is the regression of the experimental measurement. Thus, any other regressions during data processing are not appropriate; on the contrary, we need to work with the “raw” experimental data or with the mean values of the raw data. But still, they should be curves (chains of points), even as “mean curves”.

### 2.1. Material

One hyperelastic material was tested in the uniaxial tension test and in the equibiaxial tension test. The basic components of the rubber compound of the specimens are natural and chlorobutyl rubber. This material is used for truck tire construction and the preparation of a numerical model of the tire is the reason for its study and characterization. The complete formulation of the rubber compound is presented in Table 1.

The specimens of this one material were tested (ten in uniaxial mode and ten in equibiaxial mode).

### 2.2. Experimental Methods

The uniaxial tension test is in accordance with ISO 37 standard [24]. The equibiaxial tension test based on the bubble inflation technique was applied [5,25].

#### 2.2.1. Uniaxial Tension Test

**Figure 1:**
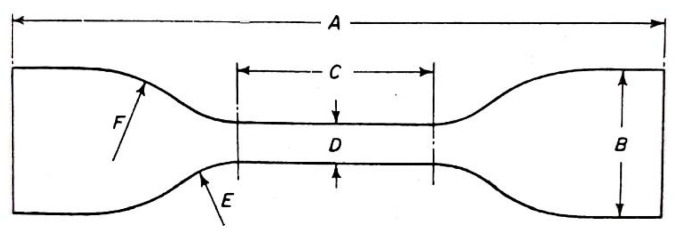
Shape and characteristics of dumbbell test specimen (letter explanations see below in the Table 2).

The uniaxial tension test was performed at a universal testing machine (Zwick/Roell 1456). The type 1 dumbbell specimens according to ISO 37 [24] were prepared (Figure 1, Table 2).

Ten specimens were cut from the same uniformly thick (2 mm) sample material sheet. The specimen was clamped at the ends and uniaxial loading at a speed of 500 mm/min was applied. The engineering stress and strain were computed from the applied force and elongation as follows:

*σ* = *F*/*S*, (1)

and

*ε* = (*l* − *l*_0_)/*l*_0_, (2)

where *σ* is the engineering stress, *F* the applied force, *S* the cross section area of the unloaded test specimen, *ε* the strain, *l* the deformed length, and *l*_0_ the initial undeformed length.

The tested specimen is mounted between two pneumatic grips of the universal tensile testing machine using compressed air. According to ISO 37 [24], the standard grip separation rate is set to 500 mm.min^−1^. As the grips move away from each other, the force is measured by the load cell, and the stress is computed according to Equation (1).

To measure the strain, the extensometer is mounted on the test specimen before the test starts. As the test specimen is stretched, the extensometer grips are moving and measuring the distance. Then, the strain can be calculated according to Equation (2). The initial length for the strain measurement is set to 20 mm. Data are recorded online by the testing machine control system as the stress/strain curves in the form of chains of discrete values at time intervals of 0.05 s. All the specimens were tested up to the limit of strain: *ε* = 1.2.

#### 2.2.2. Equibiaxial Tension Test

**Figure 2:**
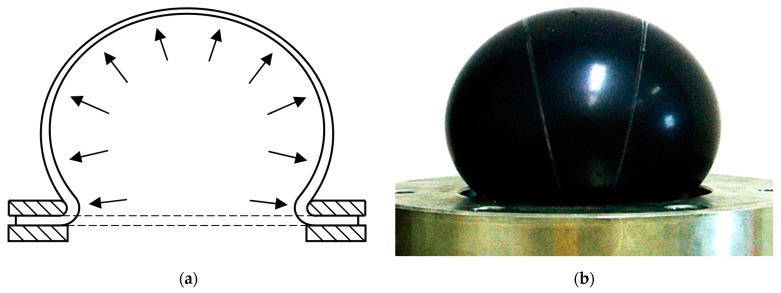
Bubble inflation technique: (**a**) schematic representation; (**b**) loaded test specimen.

There are no standards for the equibiaxial testing of elastomers. One of the methods used for this type of test is the bubble inflation technique [5,25]. A circular flat specimen clamped at the rim between two rings is inflated using compressed air (Figure 2a). The specimen is deformed to the shape of a bubble (Figure 2b).

We can continuously evaluate the stress in the material from the air pressure inside the bubble and the specimen dimensions as follows:

*σ* = (*p* × *r*)/(2 × *t*), (3)

where *σ* is the engineering stress, *p* is the inflation pressure, *r* is the curvature radius of the specimen, and *t* is the specimen thickness. The precision digital manometer (0.1% span accuracy) with a range of 0 to 600 KPa was used to measure the pressure inside the inflated specimen.

The measurement of the thickness of a deformed specimen is very complicated. With the consideration of material incompressibility, we can express the thickness as follows:

*t* = *t*_0_/*λ*^2^, (4)

where *t*_0_ is the initial thickness of the specimen. Further, we have to measure the stretch *λ* in the pole area of the inflated specimen. Generally, stretch *λ* is the ratio between the current (deformed) length *l* and the initial length *l*_0_:

*λ* = *l*_0_/*l*. (5)

Substituting Equation (4) into Equation (3) we can compute the stress *σ* as follows:

*σ* = (*p* × *r* × *λ*^2^)/(2 × *t*_0_). (6)

**Figure 3:**
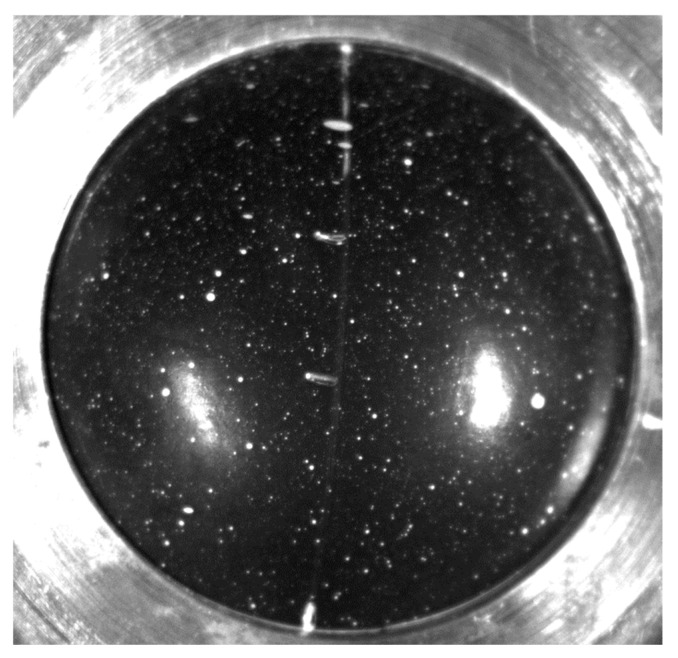
Bubble inflation technique—pattern on the test specimen.

To measure stretch *λ* on the curved surface of the inflated specimen, it is necessary to track three points in space. The digital image correlation (DIC) principle [26,27,28,29,30,31,32,33] was used for the stretch measurement. Stereo camera DIC system is able to track point displacement in a 3D space. A surface with a nonuniform color is necessary for the DIC measurement; thus, the white speckle pattern was applied on the test specimen (Figure 3).

Ten circular specimens with a diameter of 90 mm were cut from the same uniformly thick (2 mm) sample material sheet. The inner diameter of the clamping rings is 50 mm. The radius *r* and the stretch *λ* are computed from the position of the three tracked points on the bubble-shaped surface of the specimen. Then, using Equations (5) and (6), we can set up the complete stress/strain curves to describe the equibiaxial behavior of the material.

The speckled white pattern is applied on the surface of every test specimen before the testing by the spray (Figure 2). Thanks to this, three points (one in the center and the next two 5 mm on each side) are identified in the stereo DIC software. The positions of these points are tracked in the 3D space during the test and recorded by the DIC system. As the positions of points are known during the test, it is possible to determine the parameters of Equations (5) and (6), i.e., the deformed length *l* and the curvature radius of specimen *r*, and compute the stress *σ* and stretch *λ*. Then, substituting Equation (5) to Equation (2), we obtain strain as follows:

*ε* = *λ* − 1. (7)

The Mercury Real-Time tracking system was used as the control DIC system. The system is able to process input in real-time and present the results online. Two monochromatic video cameras with a resolution of 608 × 2048 pixels, pixel size of 5.5 × 5.5 μm, and 25 mm fixed focal length lenses were used. The cameras were recording the tested specimen at an angle of 20° to each other at a synchronized time rate of 0.05 s. The stress and strain values were computed by the DIC system from the inputs from the digital manometer and from cameras and then the stress/strain curves were recorded the same way as in the case of uniaxial tension in the form of chains of discrete values.

### 2.3. Experimental Data Processing and Evaluation

**Figure 4:**
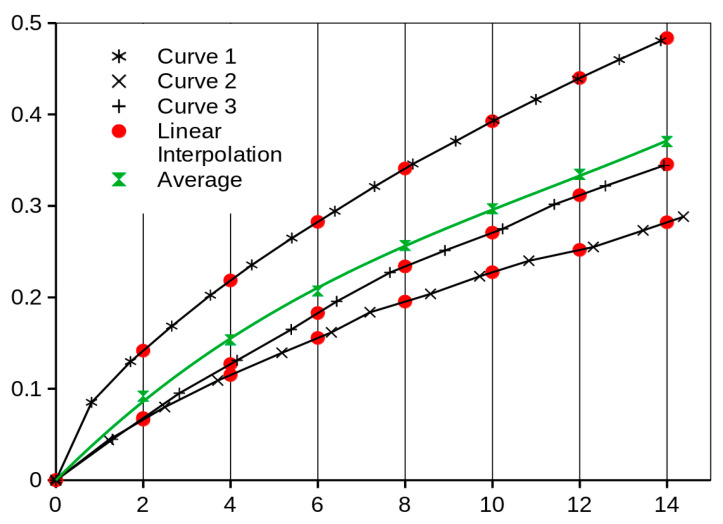
Example of the experimental data processing (in general coordinates; but the same applies to the stress/strain data)—average curve determination.

The results of the uniaxial tension test are used as the input for the prediction of the equibiaxial behavior of a material and the results of the equibiaxial tension test are used for the verification of the predicted data. Naturally, we want to use more than one specimen uniaxial test data (i.e., ten specimens in this case) to produce only one predicted equibiaxial stress/strain curve. Therefore, the first and essential problem to be solved is how to obtain only one mean curve from more measurements. The resulting curves are in the form of chains of discrete points in the stress/strain coordinates. But, the horizontal (strain axis) positions (values) of each measurement (of each point) are different (black points in Figure 4).

In the first step, the appropriate regular intervals on the horizontal (strain) axis are set up. In the example in Figure 4, they are values: 0; 2; 4; 6; 8; 10; 12; 14. In the second step, for each black curve (specimen), the linear interpolation of the stress values (vertical axis values in the example in Figure 4) of the two nearest stress values to this strain point (0; 2; 4; 6; 8; 10; 12; 14) must be performed (red dots in Figure 4). Now, we have for every strain interval point (0; 2; 4; 6; 8; 10; 12; 14) on every three black curves always three interpolated (red) values of the stress directly in the vertical row, which means that only now we can compute the average value for them, i.e., green point and whole average (green) curve, respectively.

### 2.4. Method for the Equibiaxial Data Prediction and Evaluation

**Figure 5:**
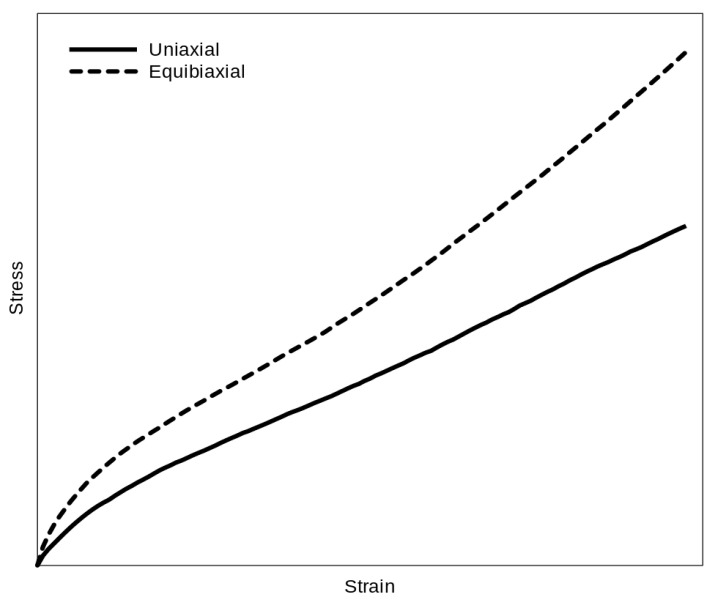
Stress/strain curve of uniaxial and equibiaxial data of common elastomer.

There is shown the difference between uniaxial and equibiaxial data for common elastomer in Figure 5. The equibiaxial values will be always higher than the uniaxial, which is evidently caused by the difference in the boundary conditions for these deformation modes. Thus, there is some relation between uniaxial and equibiaxial data for each hyperelastic material. Moreover, this relationship can be different for different of many hyperelastic materials.

The goal is to determine this relation as simply as possible. As described above, main goal of the work is to set up a relevant hyperelastic model if only uniaxial experimental data are accessible. It means that the accuracy of the relation for the computation of equibiaxial data is not the final goal, because the final equibiaxial (or any other) behavior of the examined material will be predicted by the final hyperelastic model to define which will be used in our predicted equibiaxial curve (as a function of uniaxial data). Generally, the relation should be in the form of a function:

*σ_Bp_* = *f* (*σ_U_*, *ε*), (8)

where *σ_Bp_* is the predicted equibiaxial stress curve, *σ_U_* is the experimental average uniaxial stress curve, and ε is the strain.

In a simpler form, the relation can be independent of the strain and the predicted equibiaxial stress will depend only on *σ_U_*. And further, based on the previous experiments [3,4,7,8,9] and confirmed by very close coincidence with the current experiment (presented below in the article in the Results), for most of the elastomers, this relation can be determined as a linear function:

*σ_Bp_* = *c* × *σ_U_*, (9)

where *c* is the constant ratio between the equibiaxial and uniaxial stress values. To evaluate the best value of *c* for a specific rubber compound, we will use the equibiaxial experimental data. But later, when the general values of *c* for common rubber compounds will be determined (as a material parameter), it will be possible to use only equibiaxial data to set up correct hyperelastic material constants.

If the equibiaxial experimental data are available, we can use the least squares method to find the optimal value of the *c* constant. If we are using *N* interpolation points (on the strain axis) for the function minimization, the sum of the squares of the residuals of the average experimental and predicted equibiaxial values is evaluated by the function of *c*:

*S*_(*c*)_ = Σ [(*σ_Bi_* − *c σ_Ui_*)^2^], (10)

and we are searching for the value of the *c* constant which minimizes the *S*_(*c*)_ function. Eleven interpolation points (10, 20, 30, 40, 50, 60, 70, 80, 90, 100, and 110% of strain) were used for the *c* optimization.

To evaluate the coincidence of the predicted equibiaxial data with the results of the equibiaxial experiments, the single sample Student’s *t*-test statistical method is used. Similarly, like for *c* constant optimization, only some interpolation strain points were selected for this evaluation; again, they are (10, 20, 30, 40, 50, 60, 70, 80, 90, 100, and 110% of the strain). But, it is not needed to select the same points as for *S*_(*c*)_ minimization. It is possible to test coincidence absolutely independently of the *S*_(*c*)_ minimization in the different points. In this test, the null hypothesis is that the predicted value of equibiaxial stress in the selected interpolation point is the same as the mean of the experimental values in the same interpolation point. The alternative hypothesis says that the mean and the predicted value are not the same. The *p*-values of the *t*-test are computed to evaluate the coincidence. If this value is higher than 0.05, we are not rejecting the null hypothesis that the predicted equibiaxial value and the mean of the experiment are the same.

## 3. Results

### 3.1. Uniaxial and Equibiaxial Tension Experiments

**Figure 6:**
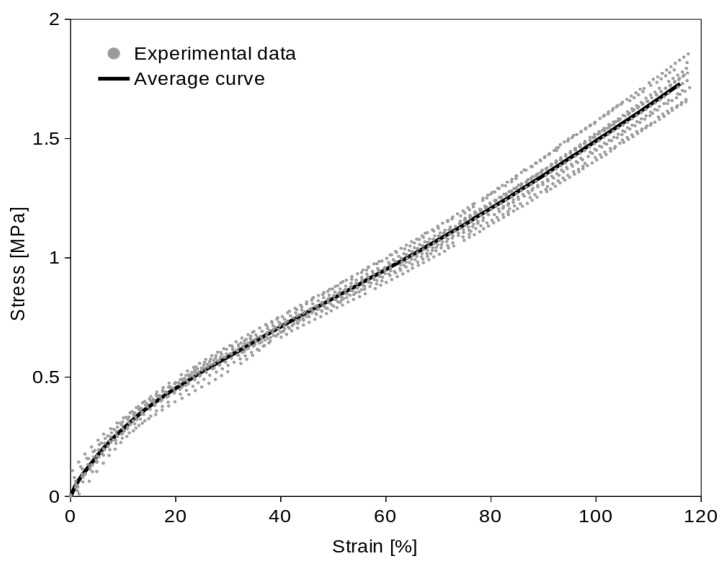
Stress/strain uniaxial experimental data (of all ten specimens) with average curve.

The uniaxial tension test experimental data of the ten specimens (of the same elastomer) together with the average curve are shown in Figure 6. The average curve was obtained by the method described in Section 2.3. The raw experimental stress data of each specimen were interpolated to every 1.0% interval of strain and from the interpolated values were computed the average points (in 1.0% strain intervals) to create an average curve.

**Figure 7:**
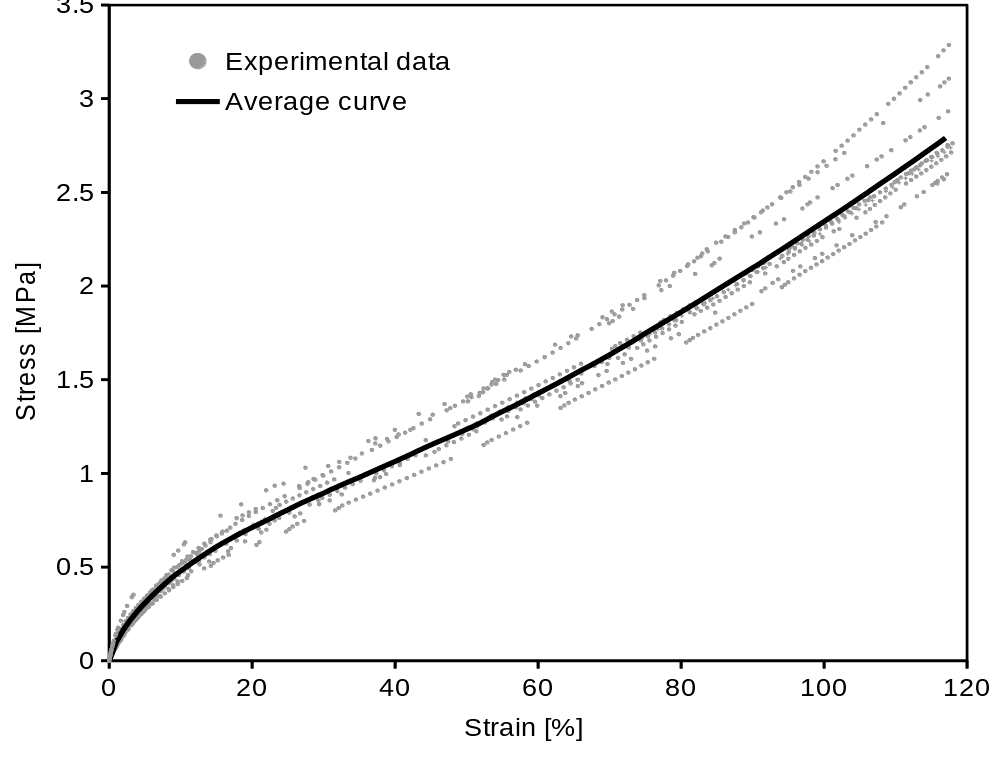
Stress/strain equibiaxial experimental data (of all ten specimens) with average curve.

The equibiaxial data, shown in Figure 7, were processed by the same method as the uniaxial.

### 3.2. Equibiaxial Data Prediction and Evaluation

**Figure 8:**
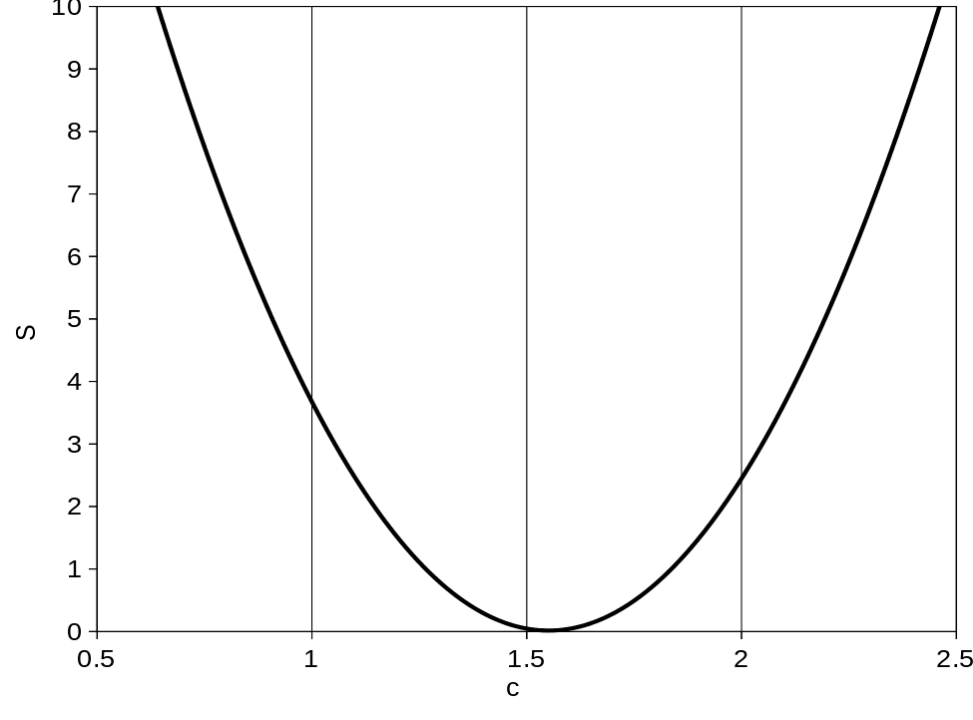
Sum of squares of residuals of experimental and predicted equibiaxial values *S* against *c* constant values.

The *c* constant for Equation (9) was computed using Equation (10). The computed sum of the squares of the residual *S* against the *c* value is shown in Figure 8.

The minimization of the sum of the squares occurs in the *c* value = 1.55. This value is valid for the material listed in Table 1 and also for the given strain range (0–120%). Substituting this constant value in Equation (9)

**Figure 9:**
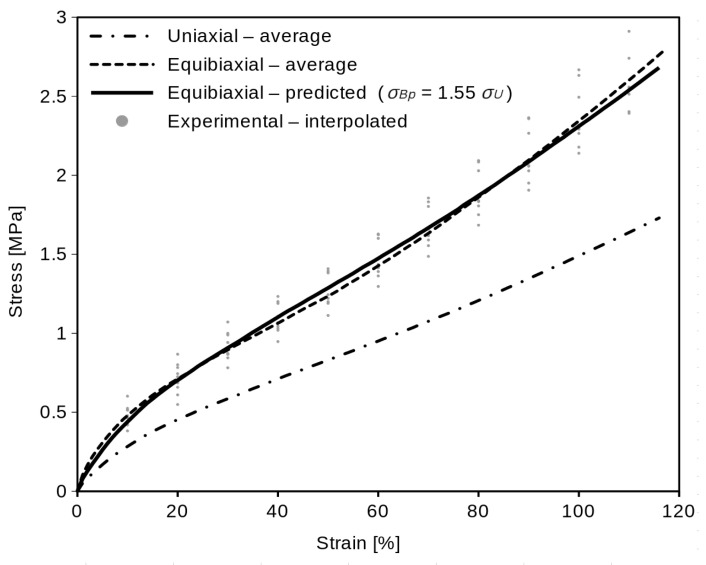
Prediction of the equibiaxial data from the average uniaxial curve.

*σ_Bp_* = 1.55 *σ_U_*, (11)

we obtain the equibiaxial predicted curve (Figure 9). Together with the predicted and average equibiaxial curve, the interpolated experimental equibiaxial data of all ten specimens for 10, 20, 30, 40, 50, 60, 70, 80, 90, 100, and 110% of strain are shown in Figure 9. The single sample Student’s *t*-test of the shown experimental points means against the predicted equibiaxial values (in each of the eleven integration points mentioned above) and the *p*-values of the *t*-test are presented in Table 3.

## 4. Discussion

The computed average curves (Figure 6 and Figure 7) show the typical “S” shape of elastomers. The optimal *c* constant value (1.55) was found for the examined material. If we use the linear function (Equation (11)) of *σ_U_* for equibiaxial data prediction, the coincidence of the predicted data with average equibiaxial data will not be absolutely exact (Figure 9), but it is appropriate enough (which is proved statistically by the Student’s *t*-test). The coincidence of the real and predicted equibiaxial data was tested in the single sample Student’s *t*-test. The *p*-values in Table 3 confirm that (at confidence level α = 0.05) we are not rejecting the null hypothesis that the predicted equibiaxial value and the mean of the experiment are the same. The high values of the *p*-values (except the 10% strain point) confirm a very close coincidence.

In spite of this sufficient result, there are more options how to predict equibiaxial data more exactly. A more complex function for the prediction could be used (Equation (8)). But, a more exact prediction is not the goal of the research. As was explained in the Introduction Section, we need to delimit the range of the equibiaxial data for the set up of the appropriate hyperelastic model in the next step (which is not the subject of this article), and finally, the coincidence of the equibiaxial curve from the hyperelastic model with real material behavior is the most important.

## 5. Conclusions

It was proved that the simple linear function of *σ_U_* can be used to predict appropriate data, usable to set up hyperelastic models even when real equibiaxial experiment data are not accessible. The method for experimental data processing, especially for creating an average stress/strain curve, was presented and the prediction function for the examined elastomer was optimized.

The main goal of the research is to be able to predict the equibiaxial behavior of elastomers only from uniaxial experiments. Therefore, the next step in the research will be searching for relations between the uniaxial/equibiaxial ratio and other generally known and usually measured properties (hardness, strength, material composition, etc.).

The next important and very useful step for the broader utilization of the presented method should also be to create a database of elastomers for which we know the uniaxial/equibiaxial relations. The described method for the mathematical formulation of prediction models and determining their constants can be applied to any available data (other elastomers presented in the literature, for example [2,3,18,19,20,21,22,23,25]) or any data measured in experimental testing. The method (the process of creating an auxiliary equibiaxial data set) is not limited by the type of elastomer, hyperelastic model, or range of loading (stress or strain). These are only the limitations for the value of the final constant of the prediction model.

Investigation as many other elastomers as possible should follow this initial investigation of ours, which could lead to the generalization of mathematical formulation and its constants, i.e., determining the limits of its values and the elimination of the limitations described above.

## Figures and Tables

**Table 1:** Recipe of tested material.

Ingredients	[phr]
NR (KLKRP, Kuala Lumpur, Malaysia)	30
CIIR (Exxon^TM^, Spring, TX, USA)	70
N660 carbon black (CABOT, Boston, MA, USA)	50
ZnO (WIEHART, Wulzeshofen, Austria)	5
Stearic acid (Evonik, Essen, Germany)	2
RAE oil (PARAMO, Pardubice, Czech Republic)	5
6PPD (Richon, Dalian, China)	2
Sulfur OT33 (Vennok^®^, Shanghai, China)	2

**Table 2:** Characteristics of test specimen type 1—according to ISO 37 [24].

Characteristic	[mm]
A—Overall length (min)	115
B—Width of ends	25 ± 1
C—Length of narrow portion	33 ± 2
D—Width of narrow portion	6.2 ± 0.2
E—Transition radius outside	14 ± 1
F—Transition radius inside	25 ± 2

**Table 3:** The *p*-values of the *t*-test of the experimental and predicted equibiaxial data.

Strain Point [%]	Equibiaxial Average Stress [MPa]	Coefficient of Variation	Equibiaxial Predicted Stress [MPa]	*p*-Value
10	0.479	0.130	0.440	0.074
20	0.711	0.132	0.703	0.777
30	0.895	0.126	0.908	0.725
40	1.064	0.122	1.102	0.372
50	1.235	0.120	1.287	0.300
60	1.428	0.122	1.474	0.420
70	1.633	0.104	1.668	0.530
80	1.861	0.087	1.872	0.831
90	2.097	0.083	2.086	0.844
100	2.344	0.081	2.310	0.583
110	2.602	0.083	2.539	0.382

## Data Availability

Data are contained within the article.
